# Phase mask-based multimodal superresolution microscopy

**DOI:** 10.3390/photonics4030039

**Published:** 2017-07-06

**Authors:** Ryan Beams, Jeremiah W. Woodcock, Jeffrey W. Gilman, Stephan J. Stranick

**Affiliations:** 1 Material Measurement Laboratory, National Institute of Standards and Technology, 100 Bureau Drive, MD, 20899

**Keywords:** Microscopy, Superresolution, Nonlinear microscopy

## Abstract

We demonstrate a multimodal superresolution microscopy technique based on a phase masked excitation beam in combination with spatially filtered detection. The theoretical foundation for calculating the focus from a non-paraxial beam with an arbitrary azimuthally symmetric phase mask is presented for linear and two-photon excitation processes as well as the theoretical resolution limitations. Experimentally this technique is demonstrated using two-photon luminescence from 80 nm gold particle as well as two-photon fluorescence lifetime imaging of fluorescent polystyrene beads. Finally to illustrate the versatility of this technique we acquire two-photon fluorescence lifetime, two-photon luminescence, and second harmonic images of a mixture of fluorescent molecules and 80 nm gold particles with > 120 nm resolution (*λ*/7). Since this approach exclusively relies on engineering the excitation and collection volumes, it is suitable for a wide range of scanning-based microscopies.

## 0. Introduction

Over the past few decades significant advancements have been made to overcome the spatial resolution limitations of conventional optical microscopy in order to probe nano-scale materials. The earliest example is near field scanning optical microscopy [1], which helped shift the interest of the microscopy community towards superresolution techniques. The most prominent of these techniques can be divided into two categories: localization microscopy and excitation engineering microscopy. Photoactivated localization microscopy (PALM) [2] and stochastic reconstruction microscopy (STORM) [3] are examples of localization microscopy. Excitation engineering microscopy includes techniques such as stimulated emission depletion microscopy (STED) [4] and structured illumination microscopy (SIM) [5,6]. While each of these techniques have provided important insight into materials, they each also have limitations. Most notably, PALM, STORM, and STED require fluorescent samples, although STED-like approaches have been theoretically proposed for coherent Raman scattering [7–9].

Excitation engineering superresolution techniques often rely on applying an amplitude or phase mask to the excitation beam. One example is a Toraldo-style phase mask [10]. In this case a series of concentric phase steps are applied to the excitation beam, which results in a focus with a narrowed centroid at the expense of increasing energy in the side lobes. The simplest example of this type of mask is a single *π* phase step, where the diameter of the *π* phase step determines the width of the centroid. While this type of phase mask has been extensively explored theoretically in the paraxial limit [11–15], investigating full theoretical foundation for strongly focused non-paraxial beams has been limited [16,17]. In addition, the substantial side lobes in the focus generated by the phase mask have limited the experimental realizations for superresolution imaging. The side lobe can be suppressed by exploiting the multiplicative nature of microscopy and has been demonstrated using two methods. The first utilizes multi-beam nonlinear processes such as four wave-mixing (FWM) and coherent anti-Stokes Raman scattering (CARS). A Toraldo-style phase mask is applied to one excitation beam and side lobes are removed by ensuring that the second beam only overlaps with the narrowed centroid of the phase masked beam [17]. In the second approach a spatial filter is implemented in the detection path to mitigate the contribution from the side lobes in the measured signal. This approach can be viewed as a multiplication of the excitation and the detection volumes. The simplest example of spatially filtered detection is tight confocal detection, but more advanced filters can also be used. This approach has been demonstrated for confocal microscopy, resulting in a ≈ 20% resolution improvement [18]. In addition, promising experimental and theoretical works have been carried out using amplitude masks combined with confocal detection [19,20], as well as super-oscillatory lenses [21–25], again showing an improvement in resolution. Significant research has also been invested into eigenmode decomposition using amplitude and phase masks [26–28].

In this work, we demonstrate that phase masked excitation in combination with selective detection can be utilized as a highly versatile superresolution technique that is suitable for a wide range of optical signals. In this case the side lobes are removed using spatially filtered detection, which results in artifact-free superresolution images. In comparison to previous reports using this type of technique, we have outlined the theoretical framework for calculating a non-paraxial focus from an arbitrary azimuthally symmetric phase mask for linear and two-photon (2P) excitation. We also explore the practical resolution limitations of the technique. This technique is experimentally demonstrated by acquiring two-photon luminescence (TPL) images of 80 nm spheres as well as fluorescence lifetime images of fluorescent polystyrene beads. To demonstrate the multimodal capabilities of this technique, we simultaneously acquire images from multiple optical signals with lateral resolution beyond what was previously accomplished [18]. Since this technique does not require a specific optical process, prior information about the sample, or post-processing, it is suitable for most types of scanning-based microscopy. To demonstrate the versatility of this approach we acquired second harmonic generation (SHG), two-photon fluorescence (TPF), and TPL lifetime images of the same sample with < 120 nm spatial resolution using 808 nm excitation. While all of these processes are nonlinear, this technique is suitable for linear optical processes as well.

## 1. Theory

To determine the ideal phase mask for superresolution microscopy, it is crucial to develop the theoretical frame work to calculate the resulting point-spread function (PSF). While the PSF is straight-forward to calculate in the paraxial limit, this is not valid for strongly the focused fields generated by high numerical aperture objectives used in superresolution applications. However, the formalism developed in Ref. [29] can be adapted for amplitude and phase mask calculations. This task has been addressed for amplitude masks [20,30,31], however, it has only limited applications to Toraldo-style phase masks [16,17]. While genetic algorithm have been implemented to optimize Toraldo-style phase masks in the paraxial limit [15], these calculations are insufficient for applications using strongly focused fields.

**Figure 1:**
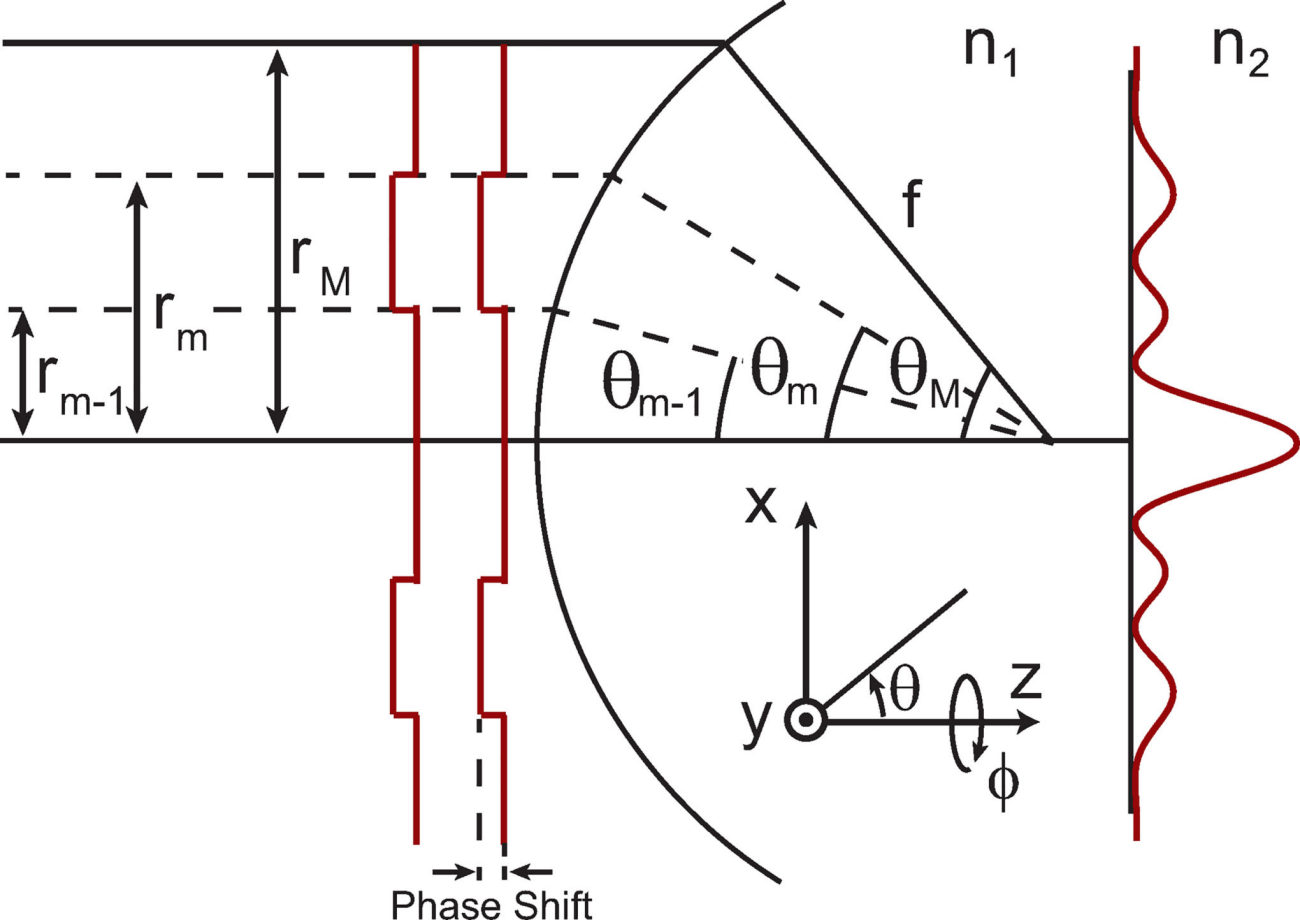
Illustrations of an excitation field being focused that has an applied phase mask. The beam diameter is *r_M_*, as defined by the microscope objective with focal length *f*. Phase mask steps are applied from *r*_*m*−1_ to *r_m_*, which corresponds to angles ranging from *θ*_*m*−1_ to *θ_m_*.

The focal fields can be calculated using the formalism for the full vectoral electric fields developed in Ref. [29]. In this case the electric fields generated by a lens with focal length, *f*, can be written as,

(1) $$\left(\begin{array}{c} E_{x}\\ E_{y}\\ E_{z} \end{array})=\frac{\text{ikf}}{2}\sqrt{\frac{n1}{n2}}E_{0}e^{-\text{ikf}}(\begin{array}{c} I_{0}+I_{2}\text{cos}2\varphi \\ I_{2}\text{sin}2\varphi \\ -2iI_{1}\text{cos}\varphi \end{array}\right)$$

where *E_x_, E_y_, E_z_* are the electric fields polarized in the *x, y*, and *z* directions, respectively, *n*_1_ and *n*_2_ are the refractive indices on either side of the interface, and *k* is the wavevector. In Equation 1, *I_i_* are defined by the integrals

(2) $$I_{\overset{0}{\underset{}{2}}}=e^{{\text{ia}}_{m}}\int_{\theta_{m-1}}^{\theta_{m}}\sqrt{\text{cos}\theta }\text{sin}\theta (1\pm \text{cos}\theta )J_{\overset{0}{\underset{}{2}}}(k\rho \text{sin}\theta )e^{\text{ikzcos}\theta }d\theta$$

(3) $$I_{1}=e^{{\text{ia}}_{m}}\int_{\theta_{m-1}}^{\theta_{m}}\sqrt{\text{cos}\theta }{\text{sin}}^{2}\theta J_{1}(k\rho \text{sin}\theta )e^{\text{ikzcos}\theta }d\theta$$

where *J*_0_, *J*_1_, and *J*_2_ are Bessel functions of the zeroth, first, and second kind. *ρ* is the radial coordinate in the focus. In Equation 2, *I*_0_ and *I*_2_ are calculated using the + and −, respectively. While similar to the integral definitions for plane waves [29], there is an additional phase term, *a_m_*, for the angular integration limits *θ*_*m*−1_ to *θ_m_* to account for the phase mask. This is conceptually illustrated in Figure 1. Finally, the total intensity in the focus can be written as,

(4) $${\left|E\right|}^{2}=\sum_{n=x,y,z}{\left|\sum_{m=1}^{M}e^{{\text{ia}}_{m}}E_{n}^{m}\right|}^{2}$$

Using Equations 1–4, strongly focused fields resulting from a phase mask with an arbitrary number of azimuthally symmetric discrete phase steps can be calculated. While Equation 4 is for a linear process, it is straight forward to extend it to nonlinear multiphoton processes. In this case the phase mask is applied to each excitation beam. For simplicity, we will restrict the discussion to linear and 2P excitation processes, where the focal fields for 2P excitation can be calculated by squaring Equation 4 to give |*E*|^4^.

**Figure 2:**
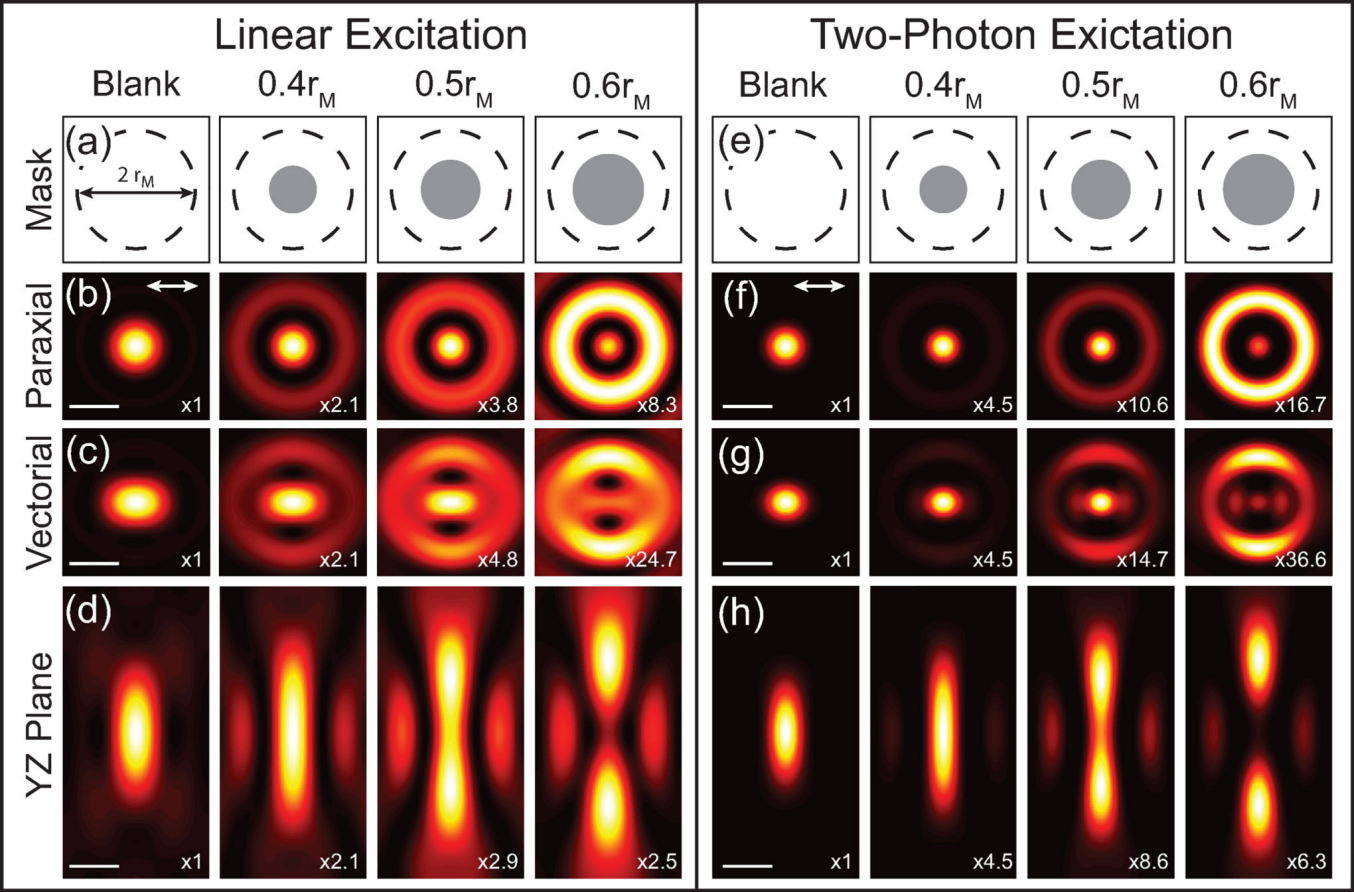
Theoretical plots of the phase masked PSF. (a),(e) Phase mask patterns applied to the SLM. The back-aperture of the objective (dashed circles) with radius r_M_ and *π* phase step applied to the SLM (gray circles) are indicated. (b)–(c) Plots of the calculations of the PSF (|*E*|^2^) in the *x*–*y* plane for a linear excitation for a paraxial and vectorial cases, respectively. (d) Vectorial PSF in the *y*–*z* plane for linear excitation. (f)–(g) PSF for 2P excitation PSF (|*E*|^4^) in the *x*–*y* plane for a paraxial and vectorial cases, respectively. (h) Vectorial PSF in the *y*–*z* plane for 2P excitation. Scale bar = *λ*/2. The direction of the excitation polarization is indicated by the double sided white arrow. The relative intensity scaling between the images with different masks is indicated.

Theoretical calculations of the focus with different phase masks are shown in Figure 2. These calculations are for a 1.4 NA oil immersion objective and a phase mask with a single *π* phase step is applied to the excitation beam with the indicated radii. The applied phase masks are illustrated in Figure 2 a,e, where the dashed line indicates the back-aperture of the microscope objective of radius *r_M_* and the gray circle is the applied *π* phase mask with the radius, *r*, indicated. Figure 2 b–d shows the calculations for the transverse intensity distribution in the paraxial limit as well as the transverse and longitudinal vectorial intensity distributions, respectively. Comparing the paraxial and vectoral calculations illustrates that the focus elongates in the direction of the incident polarization (white arrow in Figure 2 b,f) as a result of the polarization mixing from the *E_z_* component. The first column shows the case with no applied phase mask (blank), which results in a conventional focus described by an Airy function. Applying a phase mask spatial redistributes the energy in the focus and in this case results in a narrowed central lobe at the expense of increasing the intensity of the side lobes. As *r* increases, the central lobe continues to narrow and the intensity of the side lobes increase further until *r* = 0.75 *r_M_*, where the central lobe disappears. At *r* = 0.84 *r_M_* central lobe reemerges and the original Airy function is recovered for *r* = *r_M_*. Due to the polarization mixing, the central lobe also continues to elongate as *r* increases. Since the elongation is caused by the *E_z_* field components, it does not impact the focus for samples that are insensitive to *E_z_* in which case the focus closely resembles the paraxial limit. While this phase mask reduces the size of the central lobe, it also increases the longitudinal extent of the focus. Therefore the phase mask should be chosen for the specific application. While more complicated excitation masks can be used, the width of the centroid cannot be significantly reduced by going beyond a single *π* phase step [15].

The calculated PSF for 2P excitation processes using the same phase masks as the linear cases are shown in Figure 2 f–h. In this case the phase mask is applied to both excitation beams, resulting in a multiplicative effect. While these PSF calculations are for degenerative nonlinear processes where the two excitation beams are at the same wavelength, the same procedure can be applied to nondegenerative processes like four-wave mixing [17]. Comparing the 2P PSF to the linear PSF the central lobe is further narrowed and there is a greater spatial separation between the central lobe and side lobes. In addition, the side lobes are suppressed in the 2P case for phase masks that result in the central lobe having higher intensity than the side lobe, as can be seen by comparing Figure 2 c and Figure 2 g.

Figure 2 illustrates that applying a single *π* phase step can narrow the central lobe of a PSF and therefore improve the lateral resolution of an optical microscope. However for superresolution imaging applications, the side lobes need to be removed or suppressed. As discussed earlier, the side lobe can be removed by spatially filtering the signal during collection. The total PSF of the optical system is then a multiplication of the excitation and collection PSF. Therefore the detection volume can be chosen to suppress the side lobes while maintaining the narrowed central lobe. The confocal detection PSFs, including diffraction effects, can be calculated by convolving a circle function representing the pinhole in object space with the scalar PSF, which is defined as,

(5) $${\left|E_{\text{con}}\right|}^{2}={\left|\frac{\text{ikf}}{2}\sqrt{\frac{n1}{n2}}E_{0}e^{-\text{ikf}}I_{0}\right|}^{2}.$$

**Figure 3:**
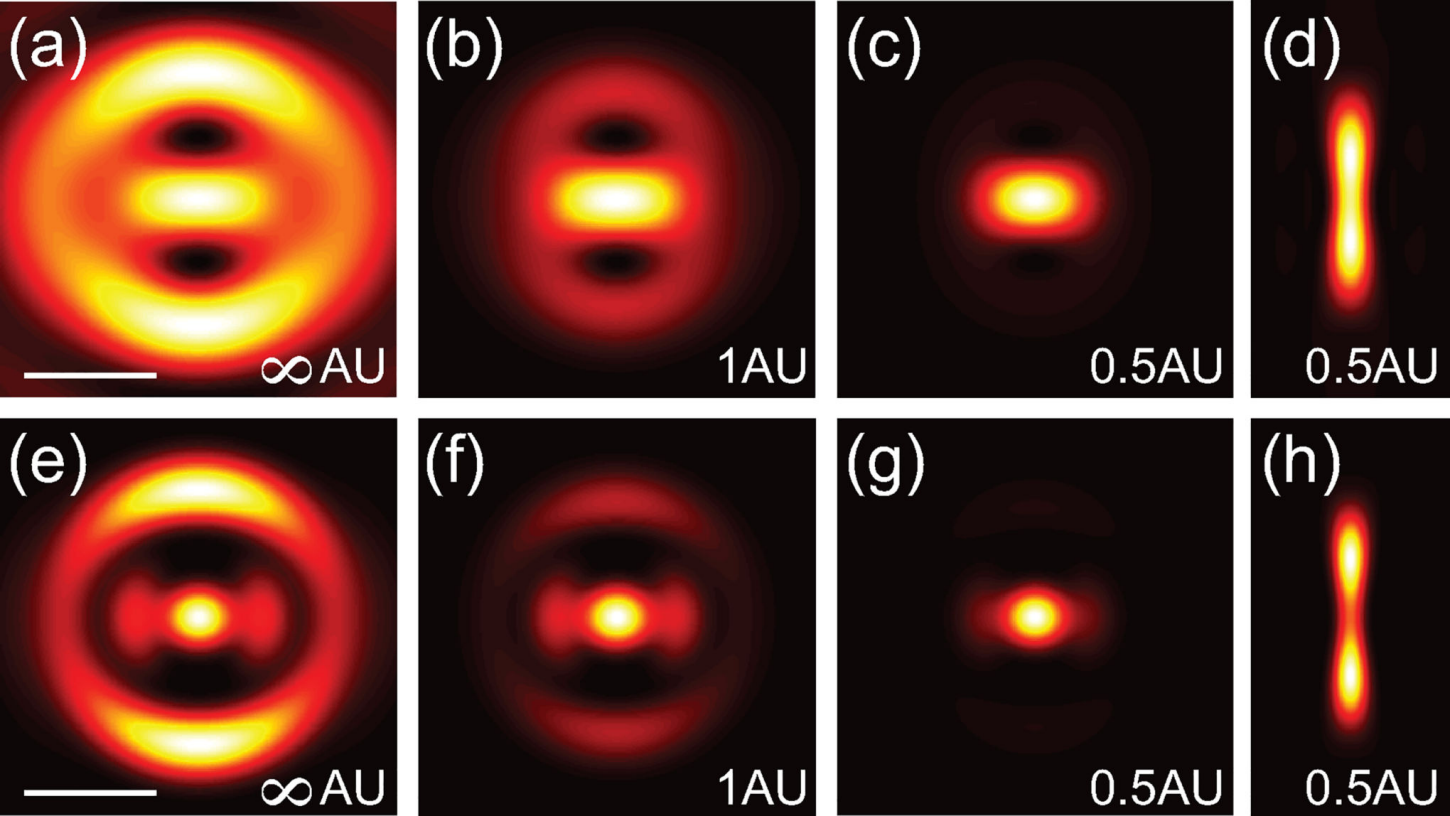
Images of the focal patterns for different confocal detection volumes for linear (top row) and 2P excitation (bottom row). PSF (|*E*|^2^) in the *x*–*y* plane with (a),(e) no confocal detection (∞ AU), (b),(f) 1 AU, (c),(g) 0.5 AU. (d),(h) PSF in the *y*–*z* plane for 0.5 AU.

**Figure 4:**
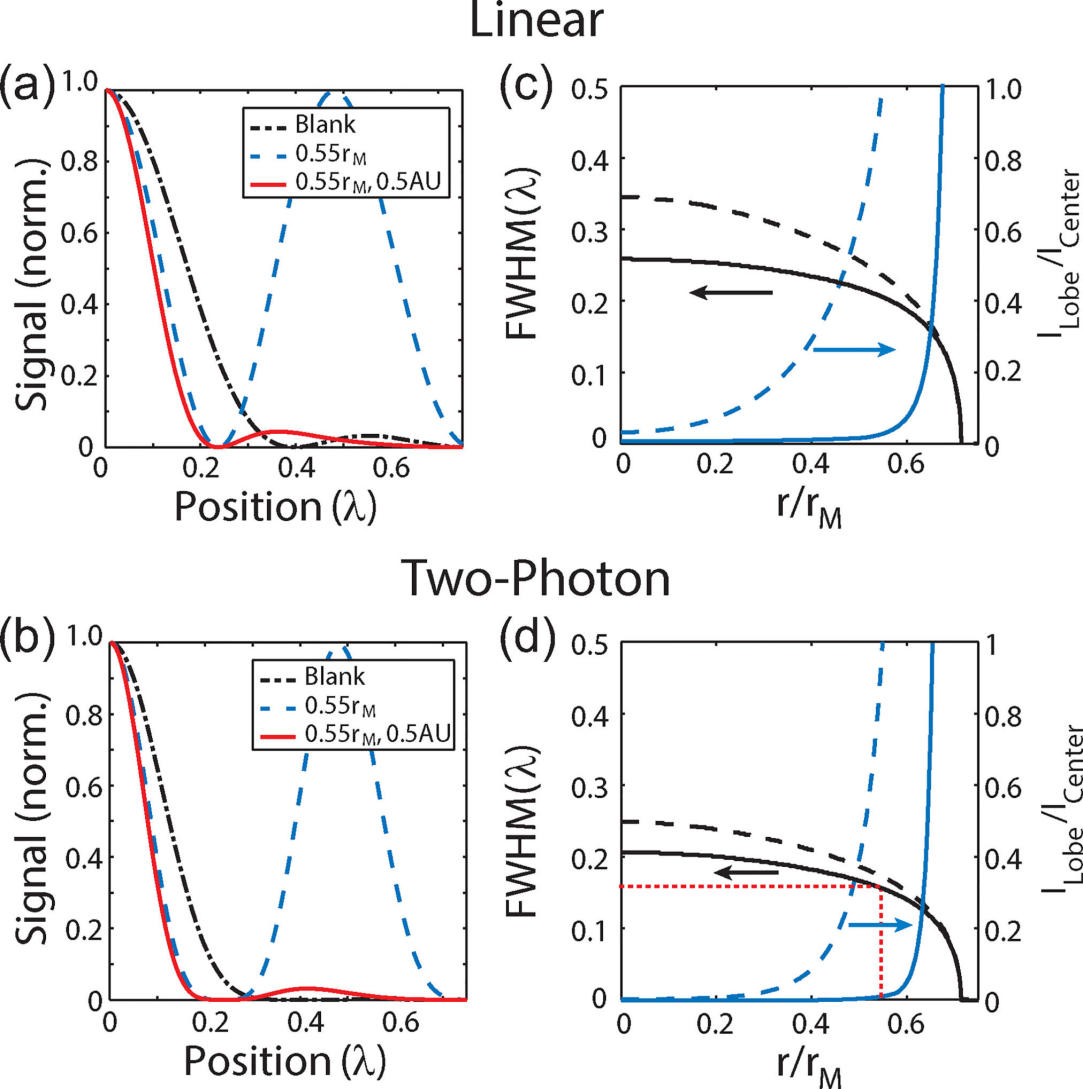
(a) Linear and (b) 2P excitation profiles of a blank (black dashed-dotted line) and 0.55r_M_ masked focus with (solid red line) and without (dashed blue line) confocal detection of 0.5 AU. (c) Linear and (d) 2P plots of the FWHM (black) and *I_Lobe_*/*I_Center_* (blue) as a function of the mask radius with (solid lines) and without (dashed lines) confocal detection.

The radius of the circle function representing the pinhole can be specified in Airy units (AU) to provide a straight forward comparison conventional confocal microscopy. In the theoretical limit for confocal detection, the circle function is replaced with a Dirac delta function, which results in a confocal detection PSF defined by Equation 5. In the case of detection, there is no polarization mixing and therefore the collection volume is azimuthally symmetric. Figure 3 a–c and Figure 3 e–g show the lateral PSFs for the linear and 2P cases, respectively, for a 0.55 r_M_ phase masked with confocal detection of ∞ AU, 1 AU and 0.5 AU. The longitudinal PSF for the linear and 2P cases are shown in Figure 3 d,h, respectively. Interestingly a pinhole of 1 AU is insufficient to fully suppress the side lobes. To more directly exhibit the resolution improvement from applying a phase-mask combined with confocal detection, Figure 4 a,b shows cross-sections of the focus for a blank phase mask (black dashed-dotted line), a 0.55r_M_ phase mask without (blue dashed line) and with confocal detection of 0.5 AU (solid red line) for the linear and 2P cases, respectively. These calculations illustrate that superresolution images can be acquired by combining a Toraldo-style phase mask with spatially filtered detection to suppress the side lobes.

While increasing the radius of the phase-mask does continue to reduce the size of the central lobe and therefore improve the resolution, it also re-distributes more of the energy into the side lobes as discussed earlier. Therefore, applications using this type of phase-mask requires balancing these two effect since the side lobes become more difficult to suppress as the strength increases and could potentially result in photo-damage to the sample. The practicality of a given phase-mask can be evaluated using the ratio of the amplitudes of the side and central lobes, *I_Lobe_*/*I_Center_*, as a metric. Plot of the full width at half maximum (FWHM) of the central lobe (black lines) on the left vertical axis and *I_Lobe_*/*I_Center_* (blue lines) on the right vertical axis as a function of *r* for the linear and 2P cases are shown in Figure 4 c,d, respectively. The cases with (solid lines) and without (dashed lines) confocal detection are shown. The red dashed lines indicate a phase mask of *r* = 0.55 r_M_ used experimentally. As these plots illustrate, while the central lobe can become arbitrarily narrow, the intensity of side lobes dominate for *r* > 0.67 r_M_ and imposes a practical limitation on the maximum phase-mask radius.

## 2. Experimental Setup and Results

**Figure 5:**
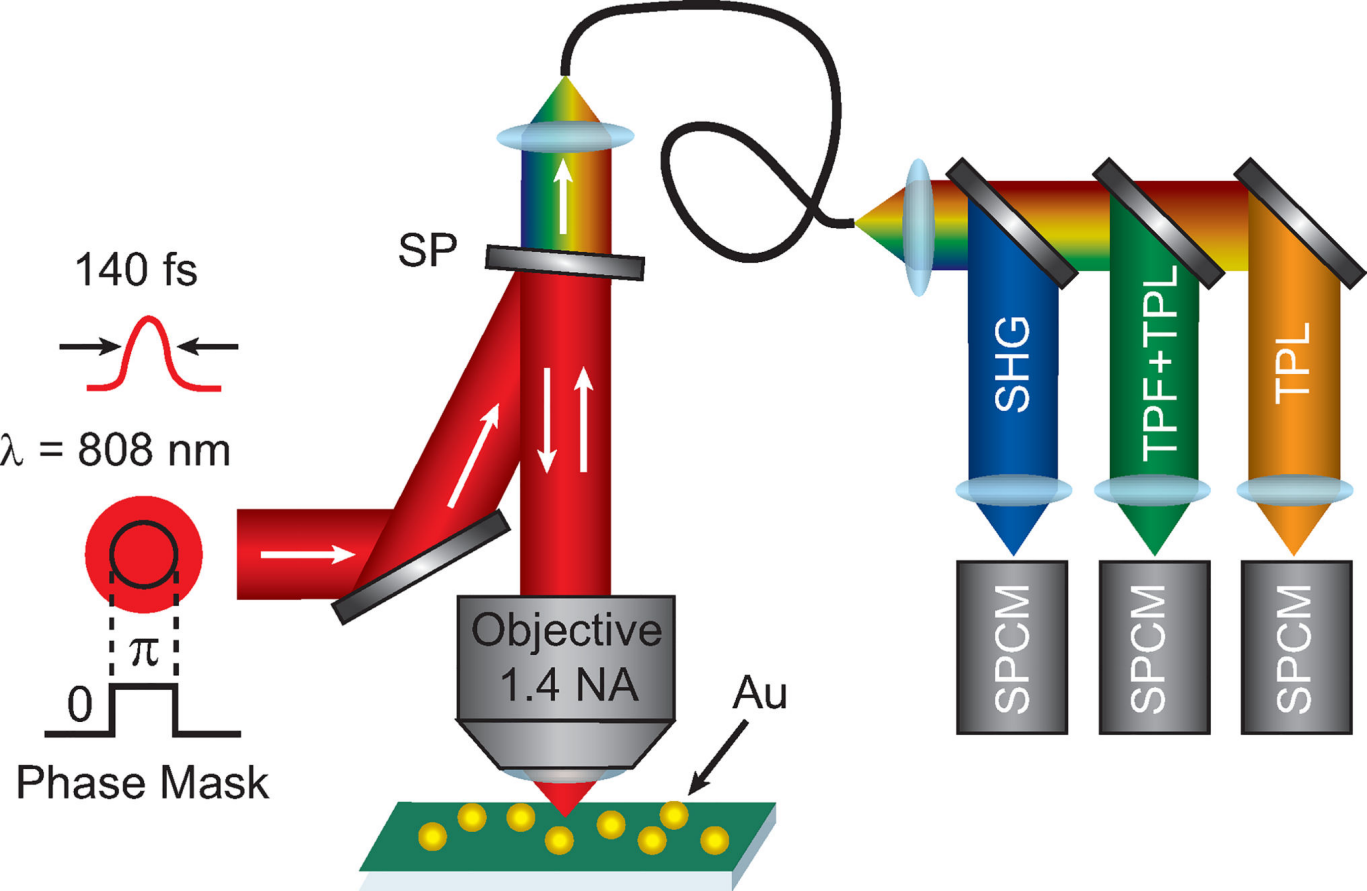
Sketch of the experimental setup. An excitation beam with a *π* phase step is focused onto the sample. The resulting signal is sent through a single mode fiber, spectrally separated using dichroic beamsplitters, and then detected on three single photon counting module (SPCM). SP = shortpass filter, SHG = second harmonic generation, TPF = two-photon fluorescence, TPL = two-photon luminescence.

Figure 5 shows a diagram of the experimental setup. A phase mask is applied using a spatial light modulator (SLM) to the excitation beam from Ti:Sapphire with ≈140 fs pulses centered at 808 nm. The applied phase mask included a *π* phase step and a background phase to remove the inherent wavefront distortion of the SLM, which was measured using a Shack-Hartmann wavefront sensor. The SLM was over-filled by a factor 2 to ensure a uniform intensity pattern across the SLM. The pattern on the SLM is imaged onto the back aperture of a high numerical aperture (1.4 NA) microscope objective using relay lenses and then focused onto the sample that is raster-scanned through the focus to create images. The resulting signal is collected with the same microscope objective and sent through a single mode fiber after passing through a shortpass filter to remove the laser excitation. The lens focusing onto the single mode fiber was chosen such that the detection area was 0.5 Airy Units (AU) for 808 nm excitation. The signal is spectrally separated using dichroic beamsplitters and sent to three time-resolved single photon counting modules (SPCM). The three SPCM detection regions as selected with bandpass filters are: 400 nm–410 nm (SHG), 420 nm–500 nm (TPF+TPL), and 555 nm–565 nm (TPL). The images presented are 256 × 256 pixels with dwell times of a 10 ms–30 ms depending on the signal levels. To compensate for the signal decrease by applying a phase mask, the incident excitation power was increased by a factor 3 compared to the blank images.

**Figure 6:**
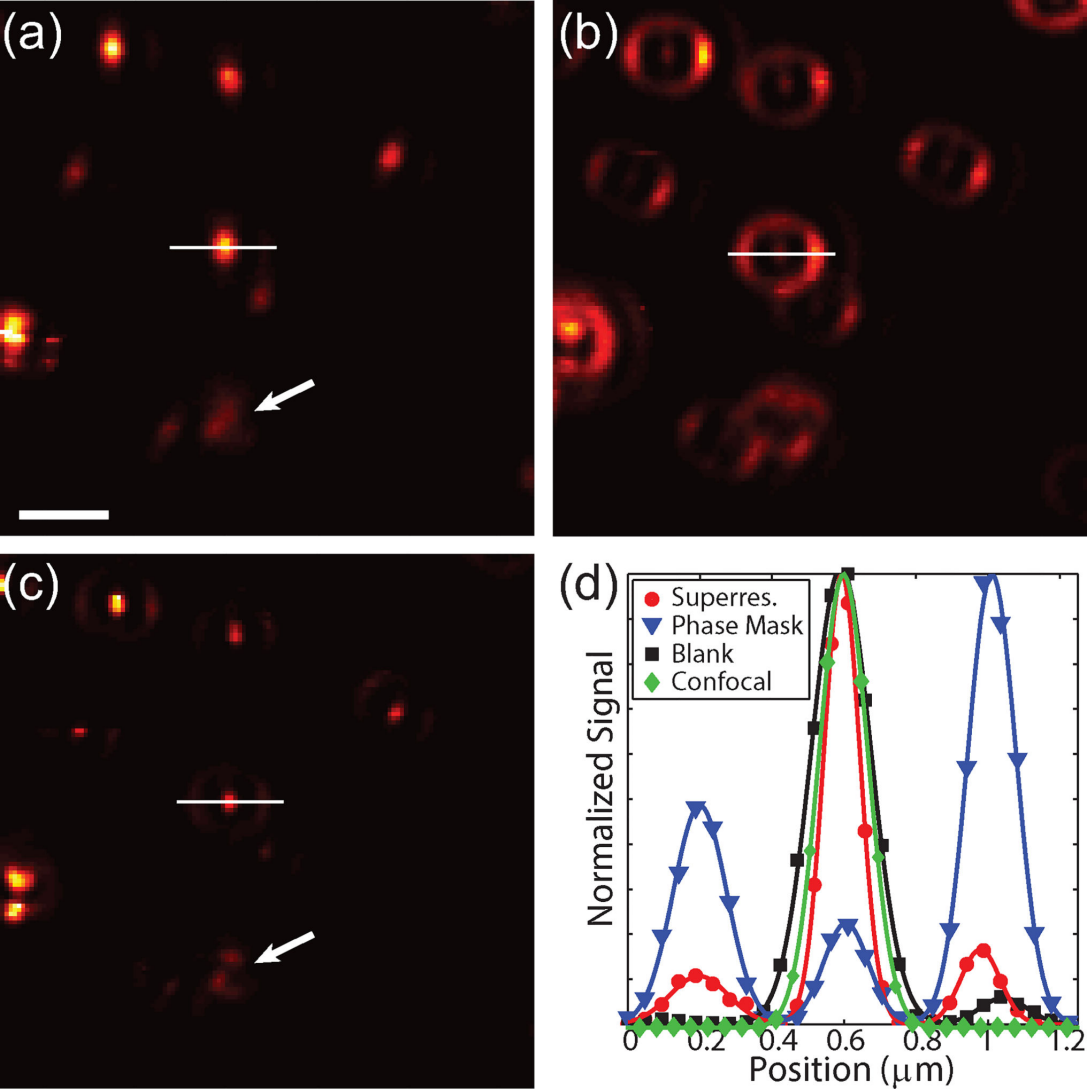
TPL images of 80 nm gold particles. (a) Blank mask. (b,c) Phase masked excitation (0.55r_M_) without and with confocal detection. (d) Normalized cross-sections along the white lines in a–c for a blank (black squares) and a 0.55r_M_ phase mask without (blue triangles) and with confocal detection (red circles). Confocal detection with a blank phase mask is shown (green diamonds) as a comparison. Solid lines are to guide the eye. Scale bar = 1 µ*m*

To experimentally demonstrate this technique, 80 nm gold spheres were drop-cast onto a glass coverslip and imaged using TPL. In this case, the photons were sent to a single SPCM. Figure 6(a) shows the conventional image with a blank phase mask and without spatially filtered detection. Applying a phase mask of 0.55r_M_ to the excitation beam without spatially filtered detection results in a narrowed centroid and increased intensity in the side lobe (Figure 6(b)), as expected from the calculations. The results from applying a phase mask and spatially filtering the detection are shown in Figure 6(c). In this case the narrow centroid remains while strongly suppressing the side lobe. Particles in close proximity can be resolved in the superresolution image, as indicated by the white arrows. Interestingly, the focal pattern is slight rotated for some of the particles, indicating sensitivity to the orientation and symmetry of the particles. For the same excitation power, the signal decreases by a factor 4 due to the reduced intensity of centroid and spatial filtered detection. Therefore the excitation power was increased by a factor 3 in the superresolution case from 0.37 mW to 1.1 mW. The increased energy in the side lobes is not a significant source of photo-damage due to the large spatial area that the lobes cover. Figure 6(d) shows cross-sections of the signal from the particle along the white lines in (a)–(c) for the blank mask (black), phase mask only (blue), and a phase mask with spatially filtered detection (red). As a comparison, confocal detection using a blank phase mask is shown in green. The full width at half maximum for the blank mask without and with confocal detection are ≈194 nm and ≈159 nm, whereas superresolution cross-section is ≈118 nm. Note that vectoral polarization mixing is seen by an elongation of the images of the particles in Figure 6(a) as well as the intensity asymmetry in the side lobe in Figure 6(b), which is in agreement with the calculations shown in Figure 2. These results experimentally demonstrate that this technique is capable of acquiring superresolution images without any post-processing or prior information about the sample.

**Figure 7:**
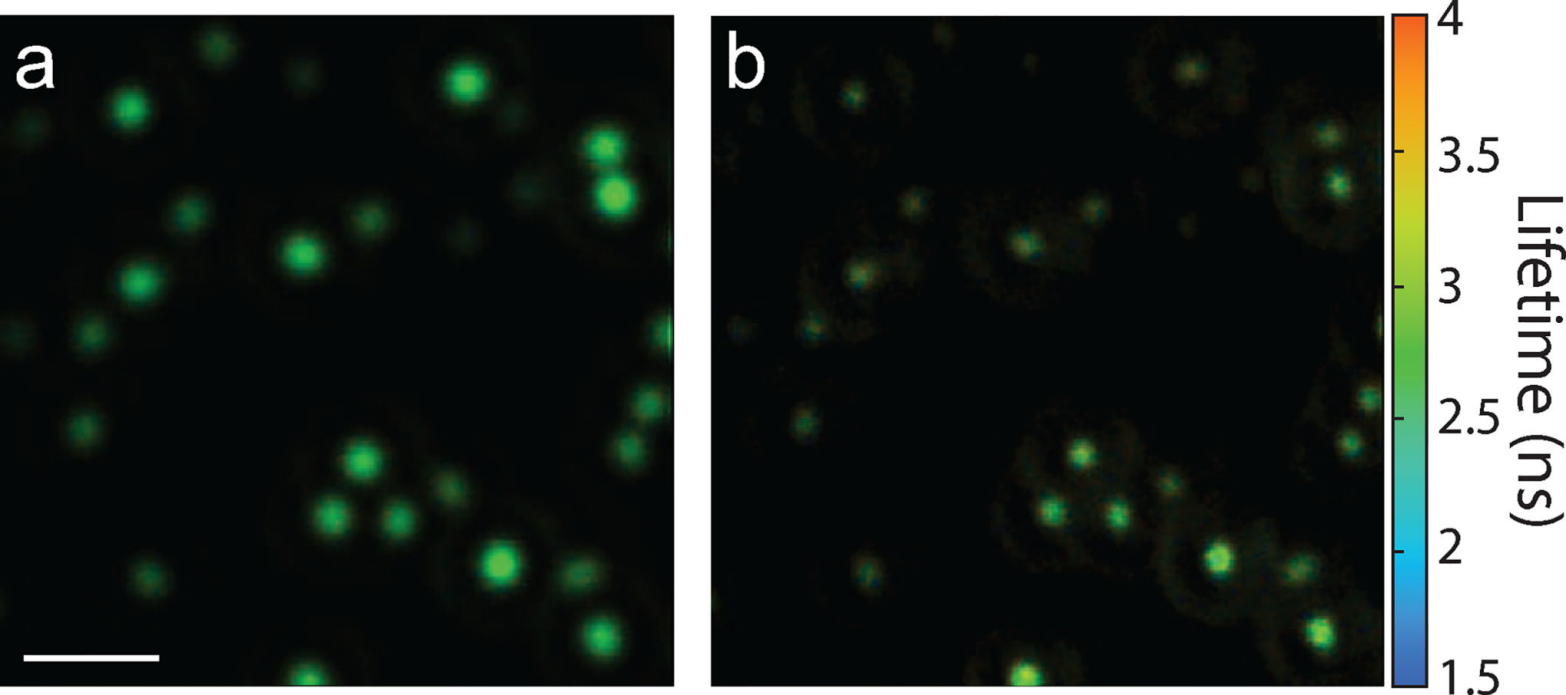
FLIM images of fluorescent beads (a) without and (b) with a phase mask *r* = 0.5 *r_M_*. Scale bar = 2 µ*m*. Color contrast scaled by ×8 in (a).

This phase-mask technique is suitable to use for a variety of optical processes. As an example, fluorescent beads (175 nm diameter) were deposited on a coverslip and the resulting fluorescence lifetime images without and with the phase mask (*r* = 0.5 *r_M_*) are shown in Figure 7 a,b, respectively. In this case, an air microscope objective (0.95 NA) was used. Comparing the two images shows a distinct resolution improvement due to the phase-mask. Furthermore, this data set demonstrates the suitability of this technique for 2P superresolution fluorescence lifetime imaging microscopy (FLIM), which has broad applications in biology.

**Figure 8:**
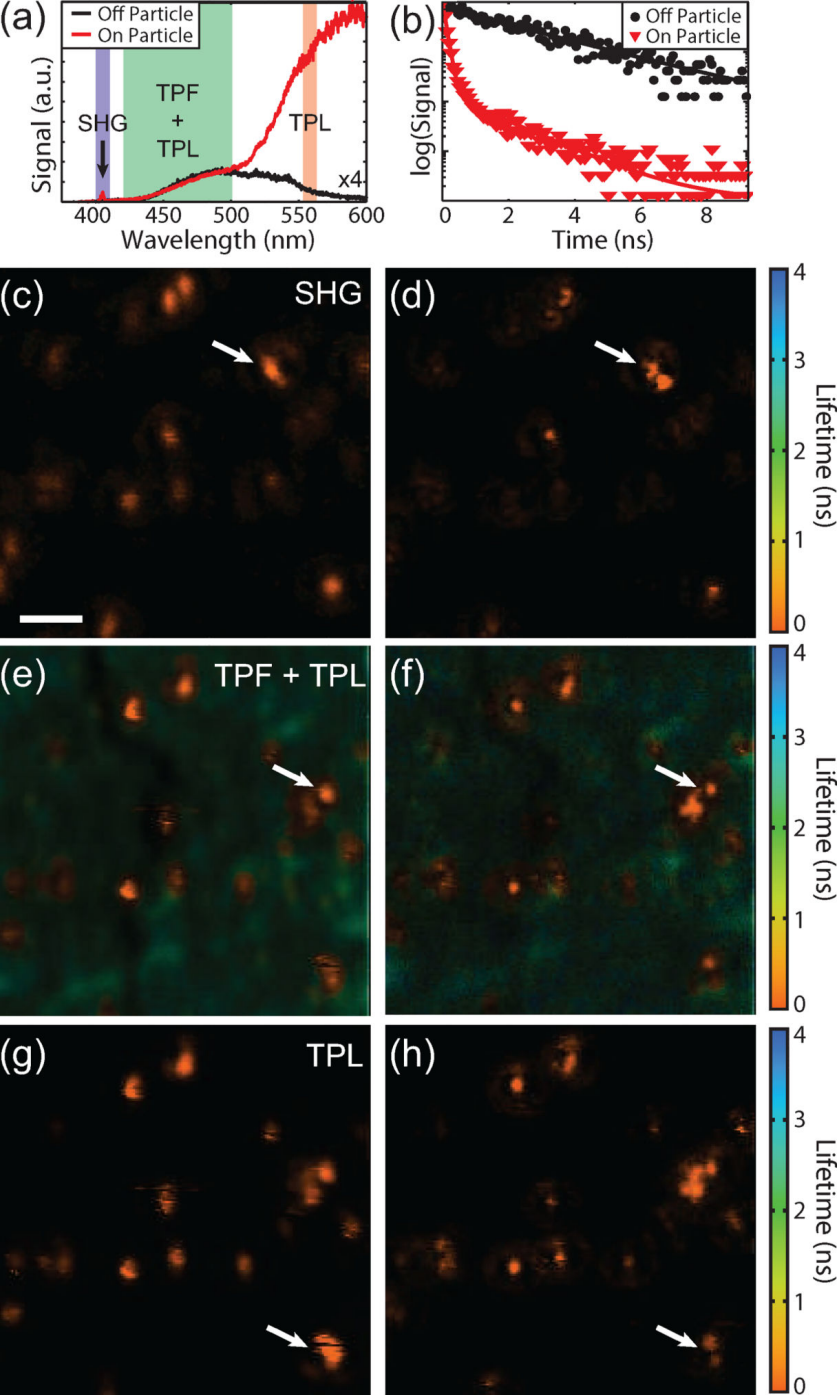
Multimodal superresolution images without (left column) and with (right column) a phase mask. (a) Spectra on (red) and off (black) a gold particle. The spectral region for each detector is indicated. The amplitude of spectra off the particle was scaled by ×4 for clarity. (b) Lifetime curves on (red) and off (black) the particle indicated by the white arrow in e. (c),(d) SHG (400 nm–410 nm). (e),(f), TPF+TPL (420 nm–500 nm). (g),(h), TPL (555 nm–565 nm). Scale bar = 1 µ*m*.

Finally to illustrate the versatility of this technique we fabricated a sample with fluorophores in addition to the 80 nm gold particles. This sample was fabricated by spin coating a concentrated (10^−5^ M) solution of coumarin derivative fluorophores onto a glass coverslip followed by 5–10 nm of poly(methyl methacrylate) (PMMA) to increase the photostability of the fluorophores. Then 80 nm gold spheres were drop-cast onto the sample. This sample was chosen because it provides three spectrally separated and unique photophysical signals. The gold particles provide SHG and TPL signals and the fluorophores provide longer lifetime TPF. Figure 8(a) shows spectra acquired on (red) and off (black) a gold particle and the detection window for each SPCM is indicated. Since our technique is scanning-based, time-resolved SPCM can be used, as has also been shown for STED [34,35]. The SHG signal from the gold particles is due to asymmetries in the shape of the particles that break the inversion symmetry [36]. The fluorophore used was selected such that the peak fluorescence is red-shifted from the SHG and blue-shifted from the TPL. The three signals were spectrally separated with dichroic beamsplitters and bandpass filters, although a fraction of the tail from the TPL is present in the TPF channel. The spectral widths of the bandpass filters were chosen to balance the relative signal levels. The fluorescence from the fluorophores can be seen on and off the particle as expected since the fluorophores were spin coated prior to drop casting the gold particles, whereas the SHG and TPL signals are only seen on the particle. In addition to spectral separation, the lifetime of the TPL and TPF are also quite different. Figure 8(b) shows lifetime decay curves on (red) and off (black) the particles indicated by the white arrow in Figure 8(e). The lifetime is longer away from the particle as expected for fluorescence. On the particle both short and longer lifetimes can be seen. The shorter lifetimes (< 0.5 ns) originate from the gold TPL as well as the fluorophores in close proximity to the gold particles.

Lifetime images of the same location for the SHG, TPF+TPL, and TPL signals are shown in Figure 8(c)–(d), (e)–(f), and (g)–(h), respectively. The left column shows the imaging with a blank phase mask and without spatially filtered detection. The right column shows superresolution images using a phase mask and spatially filtered detection. The decay curve at each pixel was fit with double exponential. The weighted average lifetime and the signal intensity are shown using the hue and saturation of the color scale, respectively. As expected, the lifetimes of the SHG and TPL from the gold particles are instrument response limited and the TPF image shows a mixture of longer lifetimes from the coumarin as well as short lifetimes from the gold. The TPF and TPL signals were acquired simultaneously with an excitation power of 0.4 mW and 1.2 mW for a blank and 0.55r_M_ phase mask, respectively. The SHG images were acquired afterward using a higher laser power due to the weak signal (1.75 mW and 5.25 mW for the blank and phase masked images, respectively). A slightly smaller phase mask (0.5r_M_) was also used due to the chromatic aberrations of the lens focusing onto the single mode fiber. Since SHG is a *χ*^(2)^ process, requiring broken inversion symmetry, and the TPL signal is a *χ*^(3)^, the differences in SHG and TPL responses/images are expected. These images demonstrate the ability of this superresolution imaging technique to measure a wide range of optical processes and acquire lifetime images.

## 3. Conclusions

We have demonstrated a versatile type of superresolution microscopy based on engineered excitation and spatially filtered detection that is suitable for a wide range of microscopy techniques. The theoretical framework presented can also be extended to more complicated phase masks as well as other excitation polarizations such as circularly polarization. While the resolution improvement is currently more modest than the established techniques, SHG and TPL images cannot be acquired using PALM, STORM, or STED. Even lifetime imaging is highly challenging for wide-field techniques due to the current limitation in detector technology. This phase mask-based approach is particularly well-suited for processes that are not limited by signal strength, such as stimulated Raman scattering and absorption microscopy, and would allow for a much greater resolution improvement.

## The following abbreviations are used in this manuscript

AU: Airy units
CARS: coherent anti-Stokes Raman scattering
FWM: four wave-mixing
FWHM: full width at half maximum
NA: numerical aperture
PSF: point-spread function
PMMA: poly(methyl methacrylate)
SHG: second harmonic generation
TPF: two-photon fluorescence
TPL: two-photon luminescence
2P: two-photon excitation
PALM: Photoactivated localization microscopy
SLM: spatial light modulator
SPCM: single photon counting module
STED: stimulated emission depletion microscopy
STORM: stochastic reconstruction microscopy
MDPI: Multidisciplinary Digital Publishing Institute
